# Factors influencing self-care management in adult hemodialysis patients: An integrative review

**DOI:** 10.5339/qmj.2024.12

**Published:** 2024-03-14

**Authors:** Fadumo Yasin, Fadi Khraim, Mark Santos, Daniel Forgrave, Abdullah Hamad

**Affiliations:** 1 Hamad Medical Corporation, Doha, Qatar Email: fyasin@hamad.qa; 2 Faculty of Nursing, Qatar University, Doha, Qatar; 3 University of Calgary, Doha, Qatar

**Keywords:** end-stage renal disease, hemodialysis, self-care management, barriers, facilitators

## Abstract

**Background:** End-stage renal disease (ESRD) poses a significant health challenge, with hemodialysis (HD) being the most prevalent therapy. Patients undergoing HD must comply with a strict therapeutic regimen, including dietary control, fluid restriction, and medication adherence. Successful disease management and improved outcomes rely on patients’ involvement and participation in their care.

**Aim:** To identify the factors that hinder or facilitate self-care management (SCM) in HD patients.

**Methodology:** This review followed Whittemore and Knafl’s integrative review framework. A comprehensive literature search of articles published between 2017 and 2022 was conducted in CINAHL, Medline, and PubMed using the keywords *end-stage renal disease*, *hemodialysis*, *self-care management*, *self-care*, and *self-management*. This search yielded 21 suitable articles for review.

**Results:** SCM is influenced by three main factors: facilitators, barriers, and outcomes. Facilitators of SCM include self-care management interventions, patient knowledge, socio-demographic factors, family support, healthcare professionals, peer support, and psychological factors. Barriers encompass psychological and physical conditions. Outcomes include both physiological and psychological aspects.

**Conclusion:** Understanding the factors influencing SCM in HD patients is vital for developing reliable and effective self-care strategies and interventions to enhance both physical and psychological outcomes.

## Introduction

End-stage renal disease (ESRD) presents a major global health challenge with high prevalence and incidence rates. Life-sustaining interventions, such as transplants, hemodialysis (HD), and peritoneal dialysis (PD), are necessary for managing ESRD. HD is the most common method of therapy, with 63% of ESRD patients in the USA and 80% in Qatar receiving it.^1,2^ However, HD sessions, typically requiring two to three regular visits per week, can impose physical and psychological burdens.^3,4^ Patients undergoing HD therapy experience a change in their well-being, and they must adhere to strict therapeutic regimens, including dietary control, fluid restriction, and medication adherence.^5,6^ Non-adherence can result in increased Interdialytic Weight Gain (IDWG), serious health deterioration, and prolonged hospital stays.^6^

Dantas et al. identified increased IDWG as an independent predictor of all-cause mortality among HD patients.^7^ Ibrahim et al. reported that 36% of HD patients were not compliant with HD treatment durations, while Goma et al. found that 55% were not compliant with dietary restrictions.^8,9^ Noncompliance with the dialysis duration and skipping HD sessions lead to hyperkalemia, the main predictor of cardiovascular death among HD patients.^10^ Developing effective self-care strategies for ESRD patients undergoing HD entails a comprehensive investigation and thorough understanding of the factors that may hinder or facilitate their implementation.

Self-care management (SCM) plays a crucial role in controlling chronic diseases and improving outcomes. It involves monitoring signs and symptoms, medication adherence, managing medical information, mental signs, and emotional signs, and minimizing the impact of the disease on patients’ preferred lifestyles.^11^ Factors influencing SCM include knowledge, self-efficacy, family support, patient psychological status, and treatment complexity.^12^ SCM practices positively impact HD patients’ physical and psychological well-being and quality of life.^13,14^ Recognizing the importance of SCM, it is prioritized in chronic disease best practice guidelines and is a critical aspect of healthcare provision in Qatar.^15^ Self-efficacy is particularly essential in SCM, enabling HD patients to acquire knowledge and implement self-care practices effectively, by which interventions should focus on improving self-efficacy.^16^ While individual studies have explored various aspects of self-care in ESRD patients, there is a lack of comprehensive synthesis in the literature. Therefore, this review aims to address this crucial gap by consolidating and critically analyzing the existing evidence on SCM in ESRD patients.

## Methodology

An integrative review was chosen as the best method to investigate the barriers and facilitators to self-care among adult HD patients. This rigorous approach allows for synthesizing empirical and theoretical studies related to the phenomenon under investigation.^17^ Furthermore, the integrative literature review can potentially inform nursing science and evidence-based practice.^17^ Five steps were followed to ensure the rigor of an integrative review: problem identification, literature search, data evaluation, data analysis, and data presentation.

### Problem Identification

Data extraction from primary research can be complex due to the infinite number of variables across studies.^17^ Hence, it is crucial to specify the purpose of the study to extract appropriate and relevant data from primary sources. Before starting the search, a guiding research question was formulated to investigate the factors influencing self-care management among ESRD patients on HD.

### Literature Search

The search was conducted with the assistance of an expert librarian to confirm a unified search approach using the relevant keywords in all databases. Studies were retrieved from three databases: Cumulative Index to Nursing and Allied Health Literature (CINAHL), Medline, and PubMed, using relevant keywords derived from the research question. The keywords were *end-stage renal disease*, *hemodialysis*, *self-care management*, *self-care*, *and self-management*. The search was limited to peer-reviewed studies published in English between January 2017 and September 2022.

### Data Evaluation

**Figure 1.**
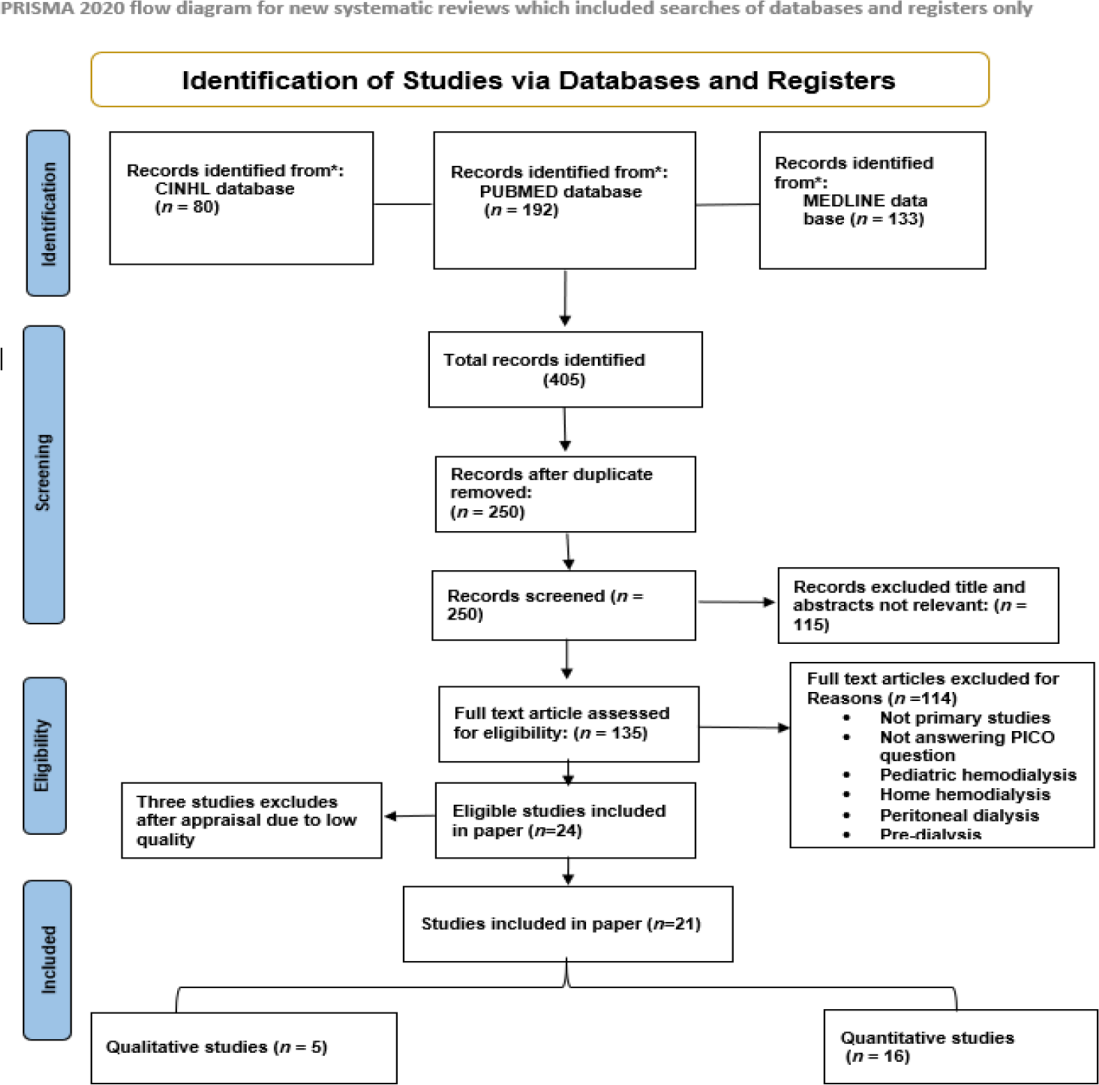
Literature search flow diagram.

A total of 405 articles were initially identified in these three databases. After removing duplicates and reviewing titles and abstracts according to inclusion and exclusion criteria (Table 1), 135 studies were selected for full-text review, resulting in 24 eligible studies. The quality of these studies was assessed using the John Hopkins Research Evidence Appraisal tool,^18^ which rates validity, reliability, and trustworthiness. Based on their methodology, the tool categorizes studies into three levels: randomized controlled trials, quasi-experimental studies, and non-experimental studies. Each article is then given a quality rating, ranging from high to significant flaws. Quantitative studies undergo a 16-question evaluation covering aspects like study clarity, validity, and reliability. Qualitative studies are assessed using a 12-question assessment focusing on the clarity of the research question and purpose, appropriateness of methods used, sample representation and adequacy through data saturation, appropriateness of data analysis strategies, and the clarity of the conclusion. Of the 24 articles reviewed, three were excluded for unclear intervention details. The remaining 21 were categorized as high (7) or good (14) quality. These comprised five qualitative, two randomized control trials, seven quasi-experimental, and seven quantitative non-experimental studies (Figure 1).

### Data Analysis

**Figure 2.**
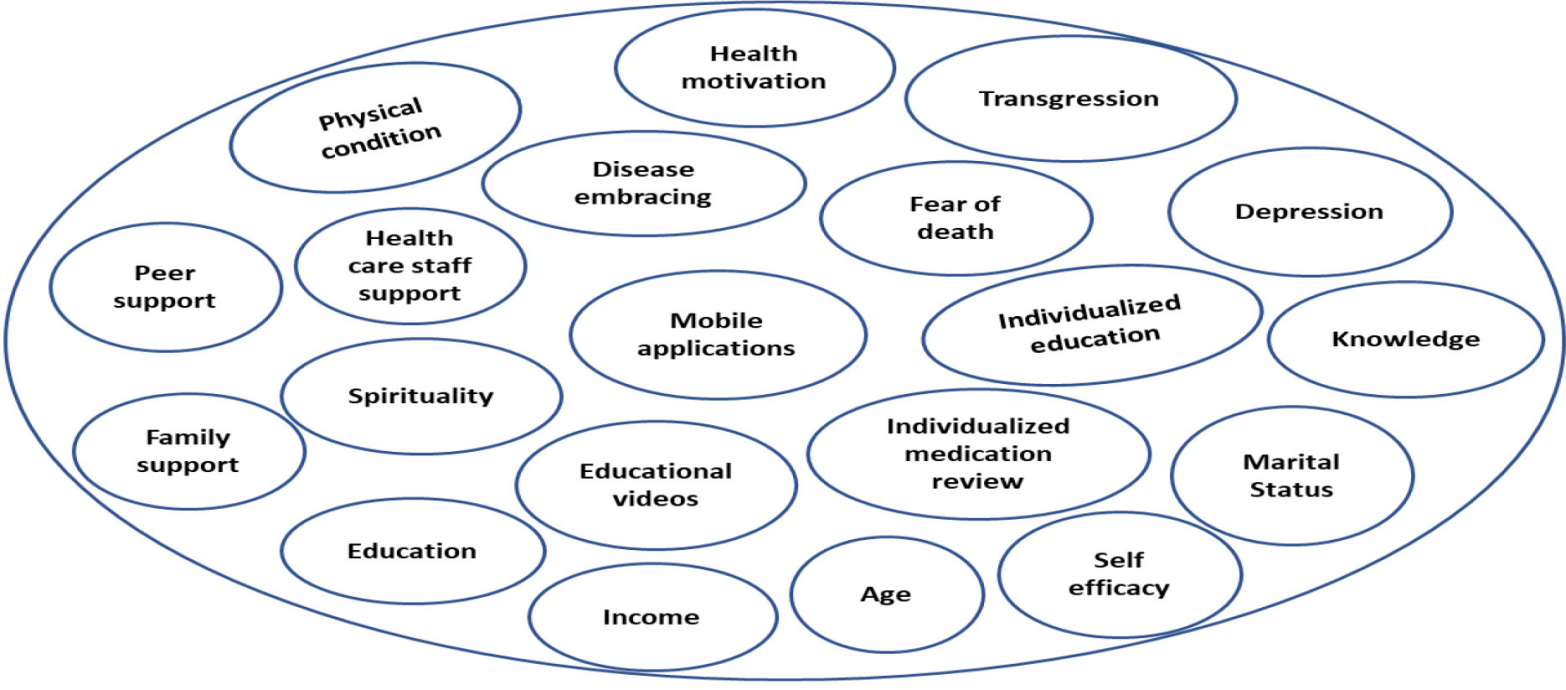
Factors influencing SCM.

**Figure 3.**
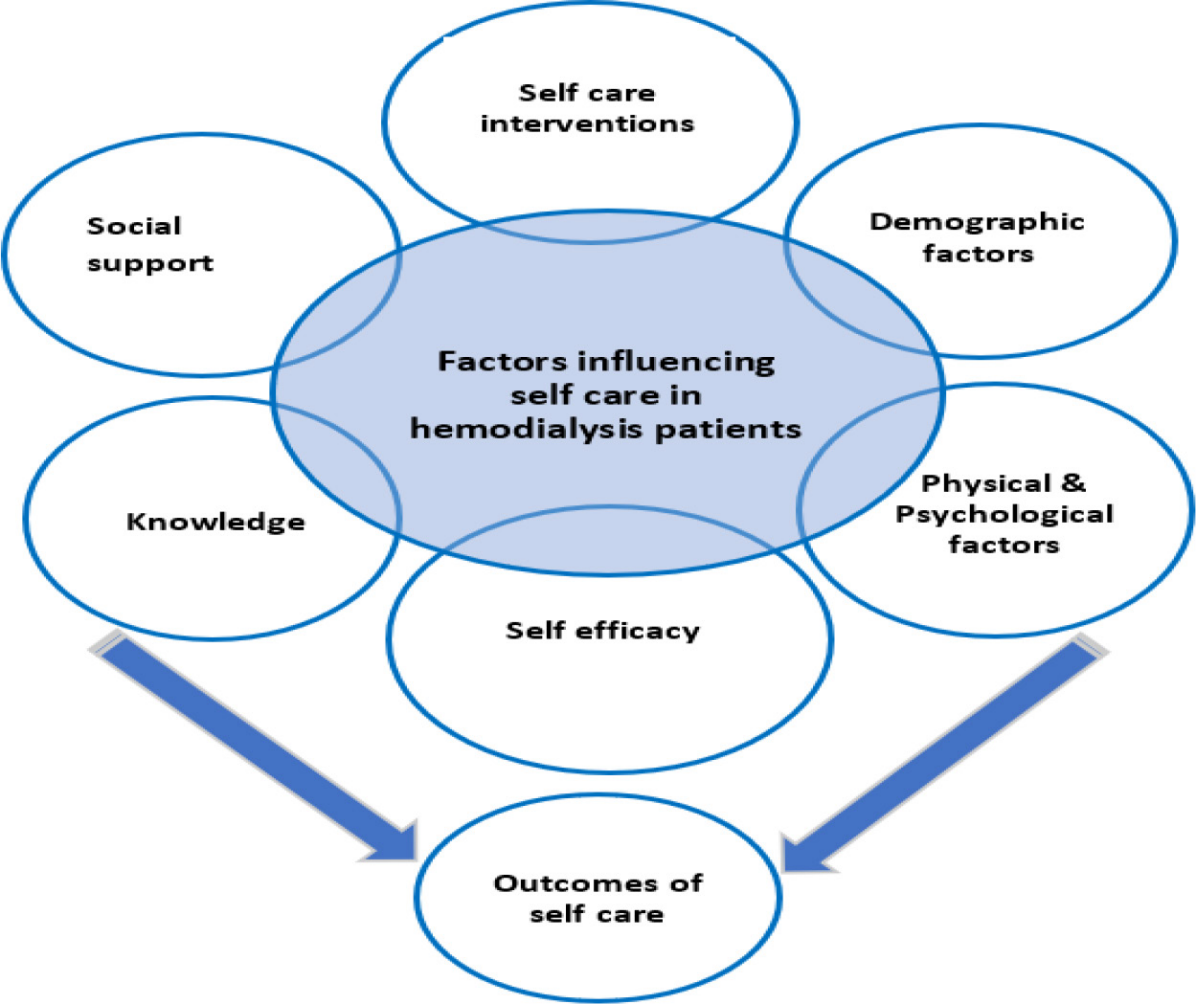
Categorization of factors influencing SCM and outcomes.

**Figure 4.**
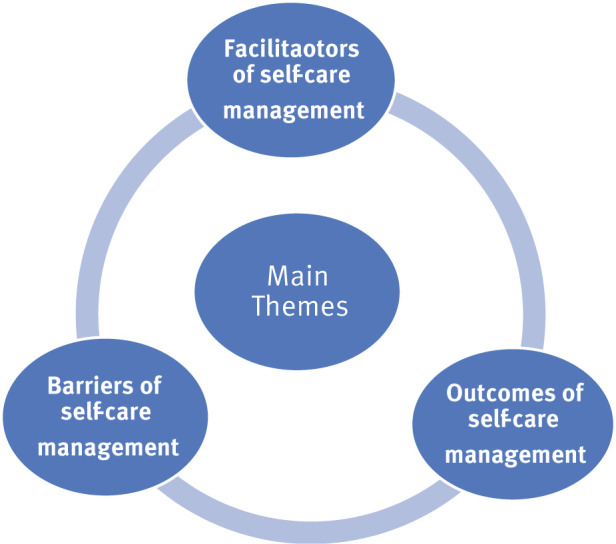
Main themes of SCM in HD patients.

Analysis of the 21 included studies followed the methodology by Whittemore and Knafl,^17^ consisting of four phases: data reduction, data display, data comparison, and data conclusion. Data from all sources were reduced and compiled into a matrix, facilitating comparison across quantitative and qualitative studies. The extraction table included the title, author, country of the research, type of study, settings, characteristics of participants, sampling criteria, data collection tools, findings, limitations, and strengths. In the data display stage, the extracted information was assembled and organized in a visual pattern (Figure 2). Then, data was compared, resulting in themes of factors influencing SCM and its outcomes (Figure 3). Conclusion drawing and verification was the last phase of data analysis, identifying commonalities and differences with each subgroup of data patterns, and three main themes were concluded (Figure 4).

## Findings

The 21 studies included in this review were conducted in Korea (*n* = 6), Indonesia (*n* = 4), Iran (*n* = 2), USA (*n* = 2), Australia (*n* = 1), Brazil (*n* = 1), Taiwan (*n* = 3), China (*n* =1), and Chile (*n* = 1). The studies included five qualitative studies, two randomized control trials, seven quasi-experimental studies, and seven quantitative non-experimental studies. Three main themes emerged from these studies: facilitators, barriers, and outcomes of SCM.

### Facilitators of SCM

The facilitators of SCM identified in this integrative review include self-care management interventions; patients’ knowledge; socio-demographic factors; family, heath workers, and peer support; and psychological factors.

#### SCM Interventions

The results of the studies included in this review showed that SCM interventions have a profound impact on HD patients’ self-management, knowledge, and skills. These interventions included using technology and health coaching.^19-29^

##### Use of Technology

Five studies included in this review have found incorporating technology into SCM interventions with HD patients effective in developing SCM skills.^19-23^ Three studies used mobile applications to introduce SCM interventions. Park and Kim evaluated a mobile application-based self-management program,^21^ Cho and Park developed a self-led educational program based on a 70-minute video,^19^ and Ren et al. examined the impact of a micro-video intervention on self-management.^23^ Two studies showed significant improvement in knowledge and self-efficacy.^19,22^ Both studies confirmed integrating smartphone applications in dietary education enhanced patients’ SCM.

##### Health Coaching

Six studies presented coaching-based interventions to improve SCM among HD patients.^24-29^ Health coaching is a practical approach to changing patients’ behaviors and enabling them to take control of their health.^30^ Two studies utilized pre-designed peer-to-peer coaching interventions focused on SCM actions, such as fluid restriction, nutritional management, HD adherence, problem-solving, and decision-making.^24,25^ A significant improvement was found in patients’ quality of life (*p* < 0.05) and self-care scores (*p* < 0.001).^24,25^ Healthcare professional-led coaching interventions have also been used to address SCM.^26-29^ The length of these interventions ranged from one to three months, with 50 to 90-minute sessions. These interventions resulted in significantly higher self-care scores^27^ as well as improved fluid restriction adherence, improved IDWG,^27-29^ medication adherence, laboratory values, vital signs, and HD attendance.^29^

#### Patients’ Knowledge

Patients’ knowledge is a key factor that influences SCM among HD patients. In this integrative review, six studies revealed that patients with good knowledge of HD demonstrate better SCM.^31-36^ Santana et al. discussed the importance of providing patients with accurate knowledge upon diagnosis of ESRD because this initial information will determine self-care actions for the rest of the patient’s journey with ESRD.^36^ Hafezieh et al. found knowledge to be a significant predictor of SCM (*p* = 0.007). In their study, knowledge was significantly associated with two critical dimensions of SCM: partnership (*r* = 0.21; *p* = 0.008) and problem-solving (*r* = 0.29; *p* < 0.001).^33^ Similarly, Lee and Noh found that SCM was positively correlated with HD-related knowledge (*r* = 0.35, *p* < 0.001).^34^ Additionally, Gimire et al.’s participants’ understanding of ESRD enabled them to comprehend their needs, understand the effectiveness of their medications, and adhere to treatment.^31^ Moreover, participants in Guerra-Guerrerro et al.’s study showed their understanding of the importance of the dialysis machine as a replacement for kidney function, resulting in a commitment to HD sessions.^32^ Park and Kim reported that patients and family caregivers who received health literacy-based self-management interventions had significantly better knowledge (*p* < 0.001), self-efficacy (*p* < 0.001), and self-management (*p* = 0.002), and decreased IDWG (*p* = 0.036).^35^

#### Socio-Demographic Factors

Socio-demographic factors are associated with SCM for individuals undergoing HD for renal failure, including income and employment status, age, marital status, and religion and spirituality.

##### Income and Employment Status

Income and employment status were this review’s most commonly reported socio-demographic factors. Employed individuals with ESRD have higher self-esteem and fewer difficulties in SCM; therefore, they have better-reported quality of life than retired and unemployed patients and significantly higher self-management scores (*p* = 0.01).^24,33^ Furthermore, high income is associated with better communication behavior, an essential predictor of SCM.^37^ Conversely, Natashia et al. showed that individuals with low income have low self-management scores (*p* < 0.001) and high IDWG (*p* < 0.05) due to the difficulty of following the recommended diet regimen and the consumption of a low-cost, imbalanced diet.^37^ In a study by Ghimire et al., participants revealed that financial constraints hampered their access to medicines and contributed to non-adherence.^30^

##### Age

Age has been found to impact SCM. Chiang et al. found that knowledge improvement was higher in patients over 60 years of age.^20^ Natashia et al. reported that age was positively related to patients’ ability to monitor health, make food choices, and exercise.^37^ However, Hafezieh et al. reported lower self-management scores among older patients (*p* = 0.009),^33^ and Ahmadzadeh et al. found an increase in the quality of life scores for older and younger patients.^24^

##### Marital Status

Marital status was found to impact SCM. Having a wife was found to impact SCM positively in two studies.^31-38^ One participant in the study conducted by Ghimire et al. mentioned that his wife prepares his medication and confirms he takes it.^30^ Siregar et al. attributed psychological support from their participants’ wives to a positive attitude and acceptance of HD.^37^ Conversely, Hafezieh et al. reported better self-management among single than among married patients (*p* = 0.01).^33^

##### Religion and Spirituality

The SCM practices done by patients suffering from HD and the management of ESRD are significantly influenced by religion and spirituality. For this review, only two articles revealed conclusions concerning Muslims, but no other religions were reported. For instance, Muslim participants in Hatthakit and Thaniwatthananon’s study discussed how prayer has impacted their ability to engage in self-care by bringing them peace, mental calmness, and spiritual upliftment.^39^ Islam, according to one participant, instructed her to keep fighting and seek medical attention. Another participant shared how hearing speeches about Islam inspired him to pursue medical care and not give up on his sickness. Siregar et al. also reported religion as a factor that facilitates SCM; their participants’ faith and firm belief in God motivated them to continue attending their HD sessions in the hopes of a quick recovery.^38^

##### Family, Health Workers, and Peer Support

Support received from family members, health workers, and peers has been found to improve self-care in HD patients. Five studies in this review reported the impact of family support on SCM.^31,32,34,38,39^ Family caregivers are key facilitators of self-care, leading to significantly higher self-care scores among HD patients (*p* = 0.038).^34^ Family members facilitate medication adherence and provide daily assistance to patients, increasing their commitment and enthusiasm toward SCM.^31,38,39^ Families also provide a sense of purpose and strong intrinsic motivation to attend HD sessions.^32,38^

Healthcare professionals are vital in aiding self-care management among HD patients. Research by Gimire et al. highlighted that a strong therapeutic relationship between patients and healthcare providers enhanced patient engagement and medication adherence.^31^ Notably, patients trusted pharmacists to organize their medications, and many developed longstanding, informal relationships with doctors, emphasizing clear communication and attention to their needs.^31^ Similarly, Saunders et al. reported that pharmacist involvement and regular consultations positively influenced medication adherence.^29^ Additionally, Santana et al. emphasized the pivotal role of nurses in patient education and the importance of patients maintaining open communication with physicians regarding their health and medications.^36^

Peer support is also an essential factor that improves the experience of coping with ESRD and performing self-care practices. Participants in Lee et al.’s study shared their experience of fluid restriction with other patients, which was an essential self-care predictor for HD patients.^27^ Participants in Santana et al.’s study considered peers’ experiences as a source of informational support.^36^ Furthermore, Hatthakit and Thaniwatthananon reported that visits from workplace colleagues and social interactions during religious events were found to be a source of happiness and positive feelings that could uplift patients’ spirits to perform their self-care activities.^39^

#### Psychological Factors

Psychological factors have a positive impact on SCM in HD patients.^31,32,34,36,39^ These facilitators include disease embracing, fear of death and desire to live, desire to be healthy, positive views and optimism, and self-efficacy.

##### Disease Embracing

Patients adopt disease-embracing to overcome limitations and the fundamental changes in their lives caused by renal failure.^32^ Patients have embraced their chronic renal failure by accepting the HD treatment and considering it a new phase in their lives, which has allowed them to continue living and learn how to rearrange their priorities in life between work, family, and treatment requirements.^32^ Participants in the study by Santana et al. described the HD treatment as “sacred,” indicating a high sense of responsibility.^36^ Participants in their study also shared the necessity of rearranging their routine to fit in the HD dialysis sessions.

##### Fear of Death and Desire to Live

Desire to live and fear of death were found in this review as motivators to adhere to treatment and carry out self-care activities. Hatthakit and Thaniwatthananon reported that participants in their study attended the HD sessions because they wished to be alive.^39^ This is congruent with Guerra-Guerrerro et al.’s findings that patients feared “blood poisoning” and death if they missed HD sessions and, therefore, committed to HD treatment.^32^ Furthermore, Ghimire et al. reported that patients complied with medications because they feared death due to non-adherence.^31^

##### Desire to be Healthy

Patients’ desire to be healthier is an intrinsic motivator to perform self-care.^31,36,39^ Lee and Noh reported that the motive to be healthy was significantly correlated with SCM (*r* = 0.61, *p* < 0. 001).^35^ Participants from Santana et al.’s study performed physical activity and exercise to improve their health and prevent deterioration.^36^ They maintained walking, jogging, and exercising on non-dialysis days to strengthen their bodies. Participants in Hatthakit and Thaniwatthananon’s study stated that being healthy allowed them to perform daily activities.^38^ These participants also reflected on health as a precious source of comfort. Therefore, they continued seeking treatment and medical advice for issues beyond their SCM.

##### Positive Views and Optimism

Positive views and optimism were found to be important psychological facilitators to cope with renal failure and continue SCM. Participants in Siregar et al.’s study expressed their gratitude for the ESRD condition and the disabling symptoms they suffered because they could appreciate the following healthy condition.^38^ Their participants felt satisfied and that life was approaching the ideal as they could return to their usual life activities despite renal failure. Likewise, participants in Guerra-Guerrerro et al.’s study adopted optimism and self-encouragement as strategies to avoid depression and to carry out needed life activities and SCM.^32^ According to Gimire et al., having positive attitudes toward treatment facilitates patients’ adherence to medication.^31^

#### Self-Efficacy

Self-efficacy is the key to patient SCM because it relates to patients’ self-confidence and abilities to perform SCM.^39^ Hafezieh et al. assessed the relationship between SCM, knowledge, and self-efficacy among Iranian patients undergoing HD.^33^ These researchers reported that self-efficacy was positively correlated with three dimensions of self-management: patient partnership, problem-solving, and emotional management. Moreover, Lee and Noh found self-efficacy had a significantly greater correlation with SCM (*r* = 0.72, *p* < 0.001) compared to knowledge (*r* = 0.35, *p* < 0.001) and health motivation (*r* = 0.61, *p* < 0.001).^34^ Furthermore, Ghimire et al. and Hatthakit and Thaniwatthananon found self-efficacy was closely associated with medication management behaviors among HD patients.^31,39^ Participants in both studies understood the importance of medication timings and pill recognition for proper medication management. They changed medication timings to avoid disrupting daily routines. They used aids to help them stay organized, such as using pill boxes or placing medications in visible places so medications were taken on time and regularly.

### Barriers

Psychological and physical outcomes of ESRD hinder patients from performing self-care actions. Natashia et al. associated depression with IDWG, which is an important predictor of SCM.^37^ Furthermore, Santana et al. reported deliberate non-adherence to self-care actions or treatment transgression in fluid intake, diet, and medications as a barrier.^36^ Participants in their study drank more fluids than allowed and ate the food they wished because they knew that their bodies would be stabilized the next day during dialysis.^36^ The physical condition is another barrier to SCM. According to Santana et al., patients have difficulty performing self-care actions and physical activities such as walking due to tiredness particularly on the day of dialysis.^36^ Furthermore, the severity of symptoms was reported by Gimire et al.^30^ as a barrier to medication adherence.

### Outcomes of SCM

The interventions used in the studies included in this review improved SCM. These improvements include physiological and psychological outcomes of disease control and self-management behaviors. This review’s most common outcomes were improved potassium and phosphorus levels.^20,22,24,27,29^ Three studies in this review have reported improvement in albumin levels, which indicates improved nutritional status and SCM.^20,24,29^ Moreover, hemoglobin level improvement has also been reported.^24,29^ Three studies showed improved IDWG after SCM interventions.^22,27,28^ These results are significant because IDWG is an important parameter for dialysis treatment and a self-care measure through treatment adherence and fluid restriction among HD patients. Moreover, after interventions, Saunders et al. showed an increased percentage of patients with acceptable systolic BP.^29^ The percentage of patients in their study with an acceptable systolic BP range improved from 73.8% to 91.4%, along with improved IDWG. Moreover, SCM interventions impact HD patients’ self-efficacy, knowledge, and mental health. Self-management interventions improve self-efficacy, which is a crucial mediator for knowledge and SCM.^21,22,35^ Furthermore, interventions enhance knowledge and awareness of diet and drug consumption and HD compliance.^22,24,29^ Lee et al. reported improved mental health components of patients’ health-related quality of life (*p* < 0.001),^27^ and Cho and Park reported a significant decrease in anxiety in their experimental group (*p* = 0.001).^20^

## Discussion

This integrative review described the influencing factors of SCM in HD patients. These factors were categorized into three main themes: facilitators, barriers, and outcomes.

### Facilitators

SCM interventions; patients’ knowledge; socio-demographic factors; family, heath workers, and peer support; and psychological factors faciliate SCM.

#### SCM Interventions

Technology-based and health coaching-based interventions were found to impact SCM in this review. Smartphone applications and videos were found in this review to be effective technology-based interventions that improve psychological state, health outcomes, self-efficacy, and quality of life. These findings are consistent with Dwairej and Ahmed’s findings that smartphone applications lead to more statistically significant knowledge scores (*p* = 0.005), BP control (*p* = 0.005), and self-care (*p* = 0.04).^40^ This review is consistent with previous studies in finding that smartphone applications have unique features that facilitate self-care actions and improve outcomes in HD patients. Kosa et al. reported that nutritional mobile applications greatly assist patients in tracking their food intake, recognizing food items, and estimating portions.^41^ However, Agarwal et al. did not find that using a smartphone application enhanced the self-care outcomes of diabetic patients.^42^ This disparity in the impact of smartphone applications may be due to the impractical design of the application or a lack of patient engagement and support when introducing smartphone applications.^42^

Additionally, video means were associated with improved knowledge and SCM and decreased anxiety in this review and other literature. Wang and Chiou reported that the use of multimedia CDs enhanced self-care knowledge and self-care behavior.^43^ Albert et al. reported that heart failure patients who received SCM education through videos reduced symptoms significantly and needed fewer prescriptions.^44^ Zhianfar et al. reported that the integration of videos in ESRD patients’ education decreased depression and anxiety symptoms.^45^

Health coaching was found to be an important intervention to improve self-care in HD patients. This is supported by Yangoz et al., who revealed that interventions based on coaching were influential in creating behavioral and lifestyle changes that are crucial for patients to cope with ESRD.^46^ Individualized coaching interventions should be considered because self-care requirements differ from patient to patient. Therefore, coaching duration, timing, and frequency should be tailored to the patient’s needs. Yangoz et al. also highlighted the importance of individualized coaching to improve adherence to fluid intake, diet, and medication management in HD patients.^46^ Peer-led coaching has also been found to be a practical approach to knowledge-building and emotional support in HD patient care in this review and other literature. Ferreira da Silva et al. reported the importance of peer support during the initiation of HD to facilitate comprehension of the HD process, particularly when patients are overwhelmed.^47^ Similarly, peer mentoring has positively impacted HD patients’ mental health.^48^

##### Patients’ Knowledge

Findings from this review showed that patients’ ESRD-related knowledge is a significant predictor of SCM. Li H et al. reported similar results on the positive impact of knowledge on SCM.^49^ Furthermore, knowledge leads to better self-efficacy and confidence. Therefore, patients become more than just care recipients; they become partners who show interest in understanding the care process.^50^ In this review, patients with higher knowledge scores were more engaged with healthcare professionals and asked questions about dialysis machine parameters.

Similarly, Årestedt et al. emphasized that understanding HD treatment is a mutual responsibility between patients and healthcare practitioners.^51^ Patients must observe and learn how things work to be more engaged in their treatment process. In contrast, Liu et al. found that a lack of knowledge about kidney disease and the importance of HD treatment led to missed dialysis sessions.^52^ Likewise, Rose et al. reported that a knowledge deficit among individuals with chronic heart failure was associated with non-participation in a cardiac rehabilitation program.^53^ It is important to note that patients’ knowledge is minimal upon the onset of ESRD.^51^ Many patients are either not fully alert or too ill during the first HD session due to the nature of this treatment. This reduces their ability to understand information related to their dialysis regimen and affects their comprehension of care plans. Therefore, healthcare providers must continue reinforcing the information delivered and prepare individualized care plans for each level of ESRD treatment.^51^

#### Socio-Demographic Factors

In this integrative review, income and employment status, age, marital status, religion, and spirituality were reported to influence SCM in HD patients. Higher-income was found to be an important predictor of better self-care and better quality of life for HD patients due to fewer financial challenges and increased confidence in discussing treatment alternatives. These findings are congruent with Bag and Mollaoglu’s study, which found that patients with a high income had higher self-care and higher self-efficacy.^54^ Adequate income allows better SCM regarding dietary options aligned with treatment recommendations.^55^ Additionally, higher-income individuals will have better access to care and will not miss medications and treatment.^56^ Lemos et al. reported that high-income participants showed better quality of life.^57^ In contrast, Gambin et al. reported that lower-income predicted poor quality of life.^58^

Age was found in this review to impact self-management positively or negatively, consistent with other literature. Li et al. reported that younger individuals may be more motivated to overcome the disease and may have the knowledge that helps them overcome their chronic disease relapses.^49^ In contrast, Al Salmi et al. found that younger patients skip dialysis treatment because they believe they are healthier than older patients and will not be affected by missed HD treatment.^59^ The inconsistent findings related to the influence of age on self-care outcomes in HD patients may be due to the debilitating consequences of the disease that affect both older and younger patients.^60^

Religion and spirituality, as a part of an individual’s culture, structure the individual’s beliefs about health and illness and influence the patient’s decision-making about their treatment.^61^ In this review, Muslims’ belief in God and religious practices were found to have an impact on patients dealing with end-stage renal disease and performing SCM. This is because Muslims patiently accept diseases and deal with them as a test from God, the owner of the disease and the cure.^62^ Shahgholian and Yousefi reported some patients with ESRD may ask God to heal them from ESRD and to strengthen their faith, which is a source of tolerance and coping.^63^ On the other hand, other patients with prolonged disease duration and multiple associated problems may become resentful and react with anger and disappointment to their ESRD, feeling that they do not deserve sickness and may question God about their poor health.^63^ Chatrung et al. reported that spirituality in Buddhist patients on HD was a method for them to accept ESRD and reduce anxiety and stress.^64^ These patients coped with ESRD by praying, donating to charity, and meditating. Spirituality facilitated these patients’ acceptance and commitment to treatment. Burlacu et al. reported that Christian patients with strong religious behavior, beliefs, and commitments showed higher HD treatment adherence.^65^

#### Family, Health Workers, and Peer Support

Social support was found in this review to be a key factor in supporting HD patients in coexisting with ESRD and performing self-care actions. These results are congruent with what is known in the literature about the importance of social support in SCM. Sousa et al. found social support to be a significant predictor of treatment adherence regarding medication, attendance to dialysis, follow-up, and adherence to diet and fluid restrictions.^66^ Furthermore, Chen et al. reported that family members are the primary source of tangible support because of their close involvement in patient care.^67^ Sousa et al. reported that family support becomes significant for ESRD patients because patients with ESRD have limited social relationships due to the ESRD and HD treatment requirements that limit their abilities and time for social interactions.^66^ In addition, Chen et al. found that prolonged engagement between ESRD patients and their healthcare professionals was an essential resource for self-care learning.^67^ Hazara et al. also indicated that the long-term relationship between HD patients and healthcare providers creates excellent opportunities to learn self-care skills.^68^

#### Psychological Factors

Psychological factors were found in this review to have a crucial impact on accepting ESRD and dealing with treatment requirements and life changes because ESRD imposes immense life changes that demand individuals’ inner strength to bear. This integrative review found that disease embracing is a way patients adapt to coexist with ESRD and start a new life. Ghaffari et al. also reported that disease-embracing helps to cope effectively with the prevailing situation.^69^ When individuals embrace ESRD, they are trying to give meaning to their suffering and to find suitable circumstances to wisely deal with the current issue and become acquainted with treatment requirements.^51^

Fear of death, as well as the desire to live and be healthy, were found in this review to be common responses in patients suffering from ESRD that helped them engage in SCM activities. Fear of death has also been found in the literature to impact these patients and motivate their adherence to treatment and engagement in SCM activities.^70-73^ Additionally, Mukakarangwa et al. reported that fear of death spontaneously makes patients comply with the sessions of dialysis.^74^ In another study, Mukakarangwa et al. reported that respondents adhered to HD treatment as they desired to have prolonged lives.^75^ Mukakarangwa et al. found that symptom alleviation, decreased physical burdens, and improved quality of life motivate patients to adhere to HD treatment to live a healthier, more comfortable, and near-normal life.^75^

Similarly, Collein et al. found that health status is one of the most significant factors facilitating self-care in HD patients.^76^ This review revealed that patients maintained physical activities to strengthen their bodies. Similarly, Rahimi et al. reported that physical activity is essential to improve patients’ health-related quality of life.^77^

The current review found a positive viewpoint, optimism, and self-efficacy to be important psychological facilitators to help patients cope with renal failure and continue SCM. Oliveira et al. also reported in a systematic review that a positive perception of illness was positively associated with adherence to diet restrictions, fluid control, and medication regime.^78^ Additionally, participants in Lopez-Vargas et al.’s study reported that the positive outlook on their lives enabled them to cope with ESRD.^79^ Several studies have reported that optimism enables HD patients to maintain positive thinking and attitudes toward the problem.^80-82^ Furthermore, Glover et al. observed a positive relationship between chronic kidney disease health outcomes and optimism.^83^ The complexity of the ESRD treatment regimen requires self-efficacy in SCM, which was found in this review to be an important predictor for SCM among HD patients. A systematic review by Sorat reported similar findings, which examined self-efficacy and its association with self-care.^84^ Sorat describes self-efficacy as a mediator of all SCM behaviors such as communication, partnership, and self-advocacy.^84^ Qiu et al. reported that self-efficacy was a powerful predictor of glycemic control in diabetic patients.^85^

### Barriers to Self-Care

Psychological factors were found in this review as barriers to self-care in HD patients. Similar findings were reported by Kim and Kim, who found a negative correlation between depression and self-care, as patients with high depression scores had low compliance with HD treatment.^86^ Additionally, depression was reported as a barrier to self-care in other chronic diseases such as heart failure and diabetes.^87,88^ This review reported that patients’ perceptions of HD’s impact sometimes led to transgressions. Similarly, the study conducted by Palmer et al. said that HD patients might deliberately eat and drink excessively, thinking that the next HD session would compensate for their transgression.^89^ In another study, Tovazzi and Mazzoni reported that patients construct their own understanding of fluid control. They believe that the human body comprises 70% water and that water restriction is harmful.^90^ Physical fatigue in HD patients was also found to be a barrier to performing daily activities. Limitations and inability to perform daily activities depend on the severity of fatigue.^60^ Similarly, Horigan et al.’s study participants shared that they felt lifeless and weak due to overwhelming fatigue. Hence, they were unable to achieve their daily activities.^91^

### Outcomes of Self-Care Interventions

SCM interventions in HD patients showed effectiveness in HD patients’ care and resulted in positive physiological and psychological outcomes. This is consistent with existing literature discussing interventional programs in ESRD. For instance, after implementing interventional self-management programs, Donald et al. reported physiological, cognitive, and behavioral changes in HD patients.^92^ They found an improvement in laboratory blood tests, blood pressure measures, knowledge, self-efficacy, and increased adherence to diet and medications. The psychological well-being of individuals receiving HD has also been shown to improve due to the improvement of self-care. Lin et al. reported that interventions related to diet, nutrition, and medication led to improved patient outcomes by reducing anxiety and depression.^93^ The effectiveness of the SCM interventions provides the patient with skills and strategies to modify and improve their behavior, increasing the patient’s confidence in dealing with longstanding ESRD.

## Implications and Recommendations

This review underscores the significance of structured SCM interventions in enhancing HD patients’ knowledge and self-efficacy. Integrating health coaching via technology, like smartphone apps, can amplify these benefits. Additionally, social support from family and peers is crucial for positive outcomes. Given Qatar’s diverse population, attention to the roles of religion and spirituality in SCM is vital. Healthcare providers should rigorously evaluate intervention outcomes, focusing on metrics like IDWG, medication adherence, and mental health. Further research is needed to assess the impact of these interventions on SCM behaviors and to gain deeper insights into the experiences of HD patients in Qatar for tailored care planning.

## Strengths and Limitations

This review has several strengths and limitations. An important strength is that all studies were primary sources focused on SCM in HD patients. This resulted in focused results that provided a comprehensive understanding of the factors influencing SCM among HD patients. Furthermore, using the integrative review method enabled the inclusion of quantitative experimental, non-experimental, and qualitative studies that gave a detailed description of SCM among HD patients. Another strength is that the included studies had a diverse range of participants. Having this diversity enhances the applicability of the findings in the diverse context of Qatar. Limitations of this review include convenience sampling in some quantitative studies, which may affect the generalizability of their results. Some other studies reported using self-reported measures to assess patients’ behavioral changes after the interventions, which carried the risk of bias. Another limitation is that all included articles in this review were in English, which may have eliminated literature on this topic produced in other languages. Finally, there were no studies from Qatar or neighboring Arab countries exploring SCM in HD patients.

## Conclusion

This integrative review explored the factors that influence SCM in HD patients. This integrative review revealed three main themes: facilitators, barriers, and outcomes of SCM. The identification of these factors may improve patient skills and outcomes. Integrating technology, including smartphone applications and video means, positively impacted SCM. Healthcare providers and peer-led coaching interventions were effective in improving patients’ self-care behaviors, building their abilities to self-care, and improving their self-efficacy. Social support from family members, healthcare workers, and peers positively affected SCM in HD patients. Additional facilitators of self-care included patients’ knowledge and psychological factors, including self-efficacy, optimism, having a positive view of the disease, and disease embracing. The barriers to SCM were identified as patients’ psychological and physical barriers. Identifying these barriers to self-care is crucial to developing suitable interventions that enhance SCM. Using appropriate patient-tailored self-care interventions improved HD patients’ physical and psychological outcomes. Identifying factors influencing SCM in HD patients and identifying the most effective interventions should be considered a priority in HD patients’ care.

## Conflicts of Interest

The authors have no conflicts of interest to declare.

## Figures and Tables

**Table 1.** Inclusion and exclusion criteria.

**Inclusion Criteria**	**Exclusion Criteria**
Peer-reviewed	Not answering the PICO question
Published in English from 2017-2022	Published before 2017
Adult hemodialysis dialysis population	Pediatric hemodialysis
Hemodialysis conducted in a hemodialysis center	Home hemodialysis, peritoneal dialysis, predialysis, post-transplantations
Primary studies	No clear interventions
	Secondary studies & incomplete studies
